# Supplementary material for: Thermal control of sequential on-surface transformation of a hydrocarbon molecule on a copper surface
Source: Nat Commun. 2016 Sep 13;7:12711. doi: 10.1038/ncomms12711 (PMC5027244; doi:10.1038/ncomms12711)
Supplement: Supplementary Information — Supplementary Figures 1-13, Supplementary Table 1, Supplementary Discussion and Supplementary References [file ncomms12711-s1.pdf]

Supplementary Information:

Supplementary Figures

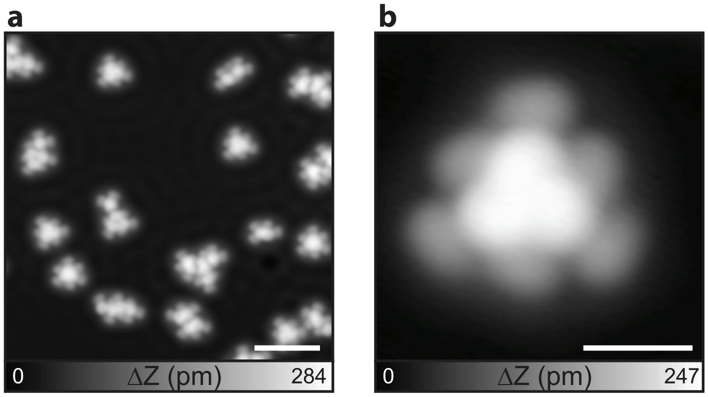

Supplementary Figure 1. | **RT deposition.** (**a**) Scanning tunneling microscopy (STM) topography of tDBA as deposited on Cu(111) at room temperature (RT). (**b**) close view of the cluster. After depositing tDBA on Cu(111) at room temperature, the sample was immediately transfered to the low temperature (LT) microscope. The overall features are the same as those observed for LT deposition (Fig. 2 of the main text), but a large triangular structure, composed of three molecules, is more frequently observed. The symmetric three-fold structure prohibits an unambiguous assignment of the individual molecules. Compared to low temperature deposition, the higher thermal energy leads to a longer diffusion length of the transformed molecule on the surface and thus they can eventually interact with each other. Measurement parameters: The tip bias voltage $V_{\mathrm{tip}} = -200$ mV and the tunneling current $I = 20$ pA in **a** and $V_{\mathrm{tip}} = -200$ mV and $I = 2$ pA in **b**. Scale bars; 4 nm in **a** and 1 nm in **b**.

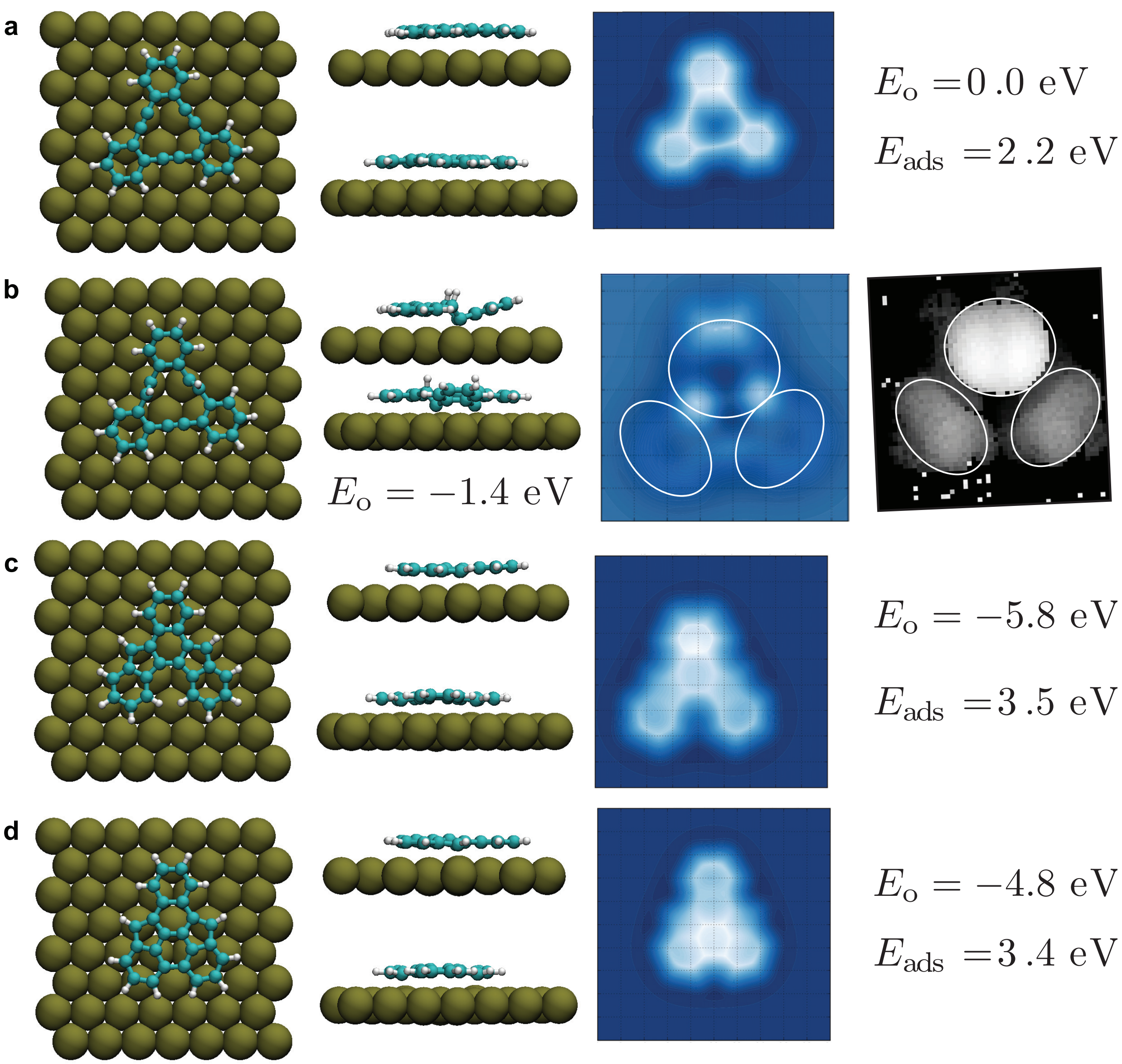

Supplementary Figure 2. (Caption next page.)

Supplementary Figure 2. | **Optimised molecular structures on Cu(111) surface.** (**a**) Intact tDBA. (**b**) First-step structure. (**c**) Second-step structure. (**d**) Third-step structure. E_{o} is the ground-state electronic energy of the complexes with respect to the intact tDBA on surface. E_{ads} are the adsorption energies – these include a basis set superposition error correction [1]. The strong interaction between the surface and the molecules is reflected in the high adsorption energies. Only the first copper layer (of six in total) is shown. The simulated atomic force microscopy (AFM) images shown in right panel, calculated using the MechAFM code (`https://github.com/SINGROUP/MechAFM/`; implementation based on the Probe Particle Model [2]). The simulated AFM image of the first transformation structure is a composite of two images taken from different heights separated by 80 pm. Similarly to the experimental AFM, the corrugation of the molecular geometry makes the simulated imaging challenging. Comparing the simulated image to the measured three-dimensional frequency shift landscape (see main text) shows that the calculated molecular geometry is able to explain the observed features: one dual-centered large bright spot (caused by the protruding H atoms) with dimmer "wings" on both sides (due to the C atoms from the still-intact triple bond and hexagonal carbon rings). Simulation parameters: The resonance frequency $f = 24.768$ kHz, the oscillation amplitude $A = 53$ pm, and the spring constant $k = 1800$ N/m.

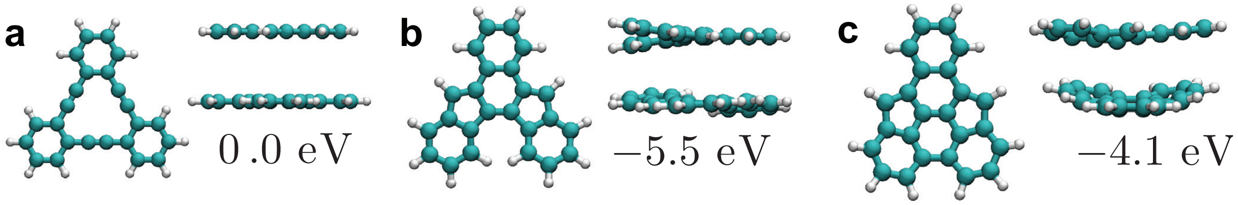

Supplementary Figure 3. | **Optimised molecular structures in vacuum.** (**a**) Intact tDBA. (**b**) Second-step structure. (**c**) Third-step structure. Ground-state electronic energies are given with respect to the intact tDBA. E_o is the ground-state electronic energy of the complexes with respect to the intact tDBA and an isolated H_2 molecule in vacuum. Note that the structure after the first reaction is not stable in vacuum, and is thus not shown here. For the first transition structure no vacuum geometry is shown as the molecular structure adapts a completely different shape in vacuum: the structure exists only on surface. A visual inspection of the structures reveals that the problem cannot be approached without explicitly taking the surface into account: the interaction with the surface changes the molecular geometries and plays an important role in the reactions.

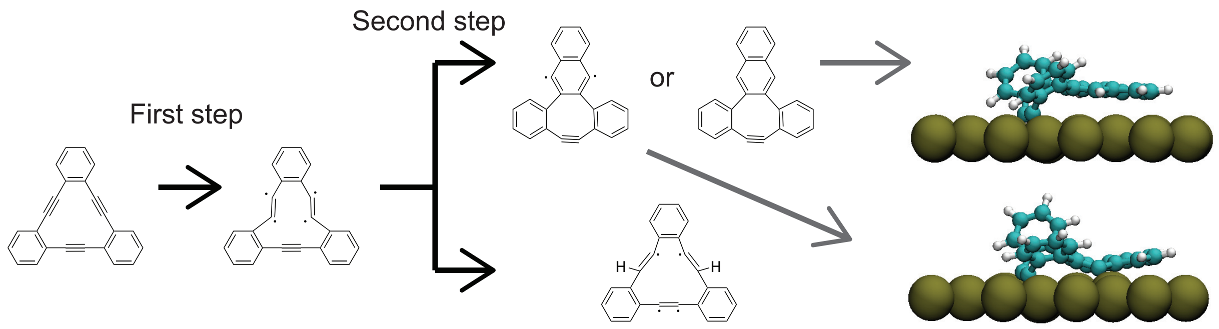

Supplementary Figure 4. | **Alternative reaction paths for the first reaction.** Based on the standard DFT computations and on the subsequent fate of the molecule, a satisfactory understanding of the transformation process on the whole was built. Note that unraveling explicitly the exact quantum chemical dynamics of how the triple bonds of the tDBA molecule are cleaved into a double bonds are unfortunately beyond the scope of this work (and are in general extremely challenging to calculate for systems as large as here). Cleaving one triple bond into a double bond exploses two non-bonded electrons – in total, the homolytic cleavage of the two triple bonds leaves the molecule with four radical centers (first step). After this, there are qualitatively two very distinct alternatives: either a naphthalene moiety is formed via new covalent C-C bond (possibly followed by hydrogen addition), or, no new C-C bonds form at this state but diffusing hydrogen atoms are introduced into the molecular structure (second step). We investigated these options using DFT structure optimisations and first-principles molecular dynamics simulations at 150 K. In the simulation run of about 2 ps, the molecule retained its geometry. The computations clearly indicate that the naphthalene moiety does not form at this stage: the molecular geometry following the newly-formed C-C bond is very corrugated and notably different from the obtained AFM image (see Fig. 2 of the main text and the molecular structures corresponding to the alternatives shown on the upper row). Furthermore, if the naphthalene moiety formed at this stage, the path leading to the second-step product (see Fig. 3 of the main text) would become very cumbersome requiring the cleaving of C-H and C-C bonds and a considerable structural rearrangement. Thus, the transformation most probably proceeds on the lower row (see the corresponding optimised structure in Supplementary Fig. 2b). It should be mentioned that there are three different possibilities where the additional two hydrogen atoms could bond. Energetically the differences among the three structures are on the order of 0.1 eV – however, only one of them (the structure considered here) leads to the observed second-step product in a straightforward manner.

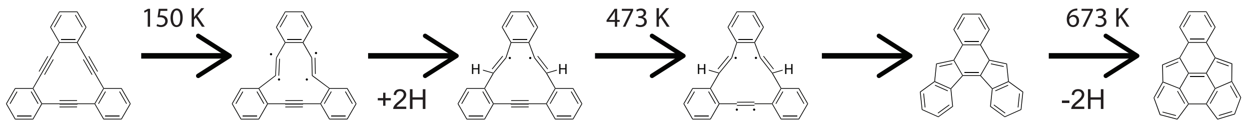

Supplementary Figure 5. | **Most likely reaction path in the tDBA transformation process on Cu(111).** With experimental analysis and theoretical analysis described in Supplementary Figure 4, the most likely reaction path is shown.

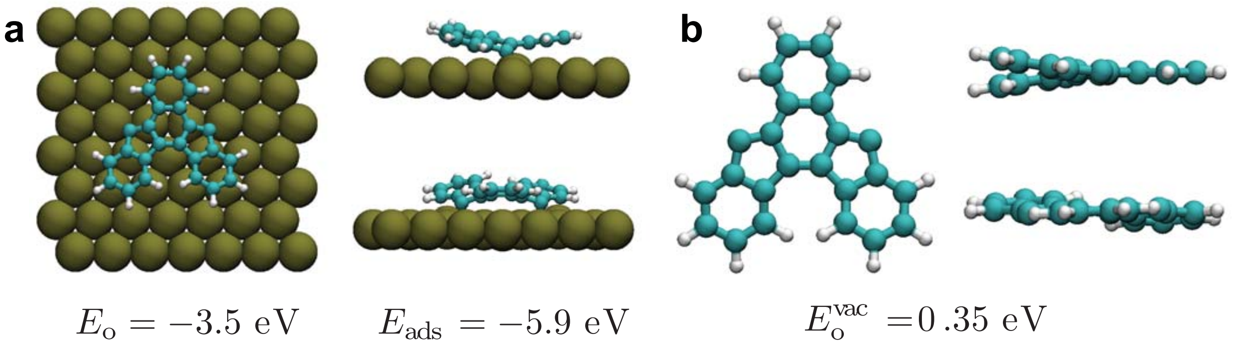

Supplementary Figure 6. | **Alternative structure for the second-step reaction.** (**a**) On-surface geometry. (**b**) Vacuum geometry. The ground-state electronic energies (E_o, E_o^{vac}) are given with respect to the intact tDBA. As discussed in the main text, in AFM imaging bonds within a molecule are less prone to be misinterpreted as bonds than bonds between molecules. In the AFM image of the molecular structure (see Fig. 3b of the main text) the two pentagonal carbon rings seem to have one hydrogen atom attached to both of them. This is an important detail, as it explicitly shows that the molecular transformation on surface involves both the addition and eventually removal of hydrogen atoms: the final structure after the third reaction contains again twelve hydrogens – based on chemical reasoning and AFM intramolecular bond interpretation (see Fig. 4b of the main text). Thus it would be of utmost interest to obtain corroborative evidence for the interpretation. To address this problem, we performed DFT calculations for the second reaction structure both with and without the additional hydrogen atoms. The molecular structure with the additional atoms ($C_{24}H_{14}$) is shown in Supplementary Fig. 2 and the version without them ($C_{24}H_{12}$) is shown here. The DFT calculations reveal that the on-surface geometries of the structures are notably different: the $C_{24}H_{12}$ interacts strongly with the surface and becomes quite corrugated; much more corrugated than the $C_{24}H_{14}$ structure. Importantly, the difference in the corrugation is large enough to be detected via AFM. In the AFM experiments the corrugation is fairly conservative: the molecule is almost planar. This indicates that the molecular structure on surface has obtained two additional hydrogen atoms during the reactions leading to this geometry; most probably the hydrogens are added already in the first reaction. This analysis supports indirectly the interpretation that the elemental composition in the final structure is $C_{24}H_{12}$ as in final structure the chemical environment of the C-H bonds is very similar to the second reaction product discussed here – this means that two hydrogen atoms are cleaved in the third reaction.

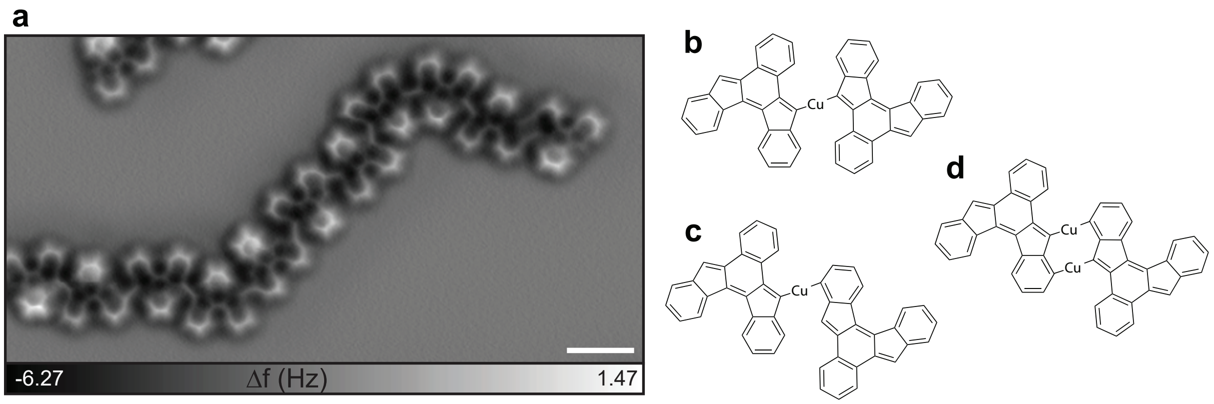

Supplementary Figure 7. | **Organometallic bond.** (**a**) Frequency shift map of the organometallic bond, formed by annealing at 200°C. (**b-d**) Schematic drawings of different chemical structures. The chemical structure of the individual unit is the same as the one in Fig. 3b of the main text, yet the transformed tDBAs are connected with each other. The gap is about 200 pm and is certainly longer than the C-C bond (134 pm). Note that the measured value is shorter than the actual length since the tilt effect of the CO tip significantly enlarges the apparent size of the benzene ring [3, 4]. Therefore, the units are likely connected via the C-Cu-C organometallic bond. The position of carbon connected to Cu is mostly at the 5-membered rings shown in **b**, but the shifted configuration shown in **c** and even double organometallic bonds shown in (d) were observed in this preparation. The structure in **b** is the dominant product. Measurement parameters: $V_{\text{tip}} = 0$ mV and $A = 60$ pm in **c**. Scale bar, 1 nm.

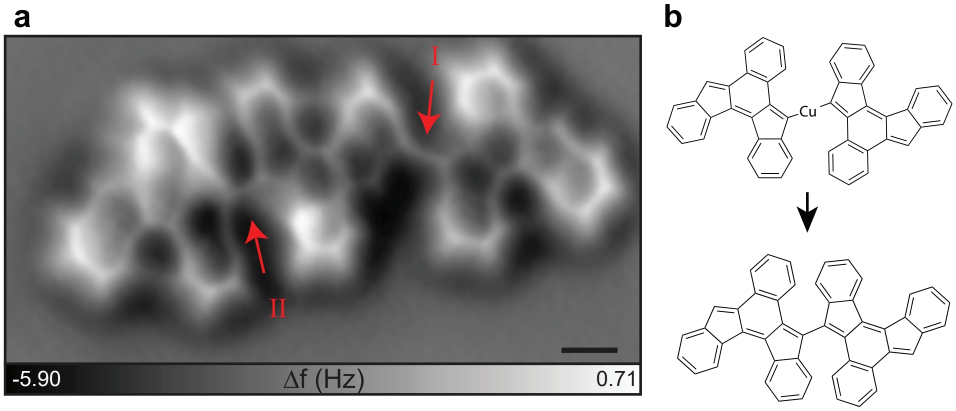

Supplementary Figure 8. | **Covalent bond in the polymer.** (**a**) Frequency shift map of the organometallic bond, formed by annealing at 200°C for 10 min. (**b**) Chemical structure of the product. Besides the organometallic bond (I), a shorter gap (II), corresponding to the C-C bond, is seen. The intermolecular gap (centre and left) is shorter, so that the the repulsion between hydrogen atoms induces the distortion in each molecules. Therefore, the top part of the left molecule and the bottom part of the centre molecule are lifted up from the surface, resulting in brighter contrasts. Nevertheless, annealing at higher than 200°C cleaves the organometallic bond. Measurement parameters: $V_{\text{tip}} = 0$ mV and $A = 60$ pm in **c**. Scale bar, 300 pm.

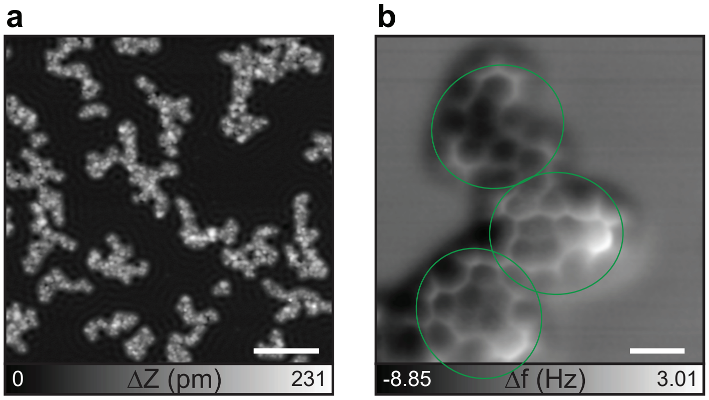

Supplementary Figure 9. | **Diffusion of carbon atom.** (**a**) STM topography of tDBA after the polymer (Supplementary Fig. 10) was annealed at 350°C for 20 min. Since all molecules in the image are connected, no single molecules are observed. (**b**) Corresponding frequency shift image, in which the molecules were fused to each other. We can see the original shape of the transformed tDBA as indicates by circles, yet some carbon atoms already occupy different configurations due to diffusion and rearrangement within the plane of the molecule. Once the organometallic bonds form, these processes cannot be avoided, resulting in small graphene flakes with an uncontrollable structure. Measurement parameters: $V_{\mathrm{tip}} = -200$ mV and $I = 1$ pA in **a** and $V_{\mathrm{tip}} = 0$ mV and $A = 60$ pm in **b**. Scale bars, 10 nm in **a** and 500 pm in **b**.

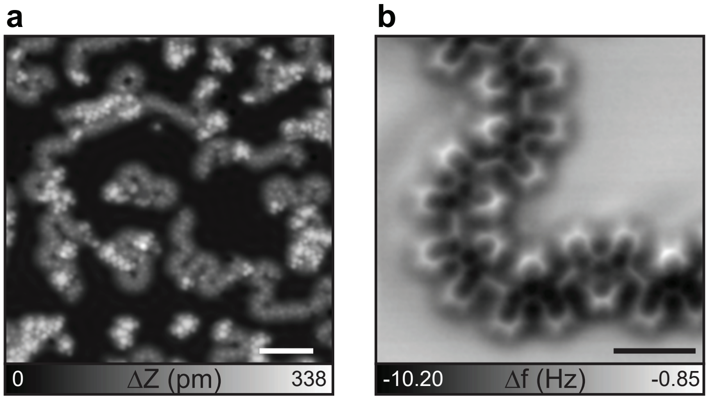

Supplementary Figure 10. | **RT annealing.** (**a**) STM topography of the tDBA, left at room temperature overnight (10 hors). Most of the transformed tDBAs were no longer isolated nor aggregated as small clusters, but formed one-dimensional polymers. Some triangular clusters are still visible at the bottom of the image, indicating that the reaction speed at room temperature is rather slow. Nevertheless, the chemical structure of most molecules changed during a night at room temperature. This observation is consistent with the fact that such one-dimensional polymers are absent in Supplementary Fig. 1. (**b**) Corresponding frequency shift image. The molecules are transformed and are the same as that obtained by annealing at 200°C. They are linked to each other via the organometallic bond, with the bond always starting at the position of carbon at the 5-membered rings shown in Supplementary Fig. 7b. Measurement parameters: $V_{\text{tip}} = -200$ mV and $I = 2$ pA in **a** and $V_{\text{tip}} = 0$ mV and $A = 60$ pm in **b**. Scale bars, 5 nm in **a** and 1 nm in **b**.

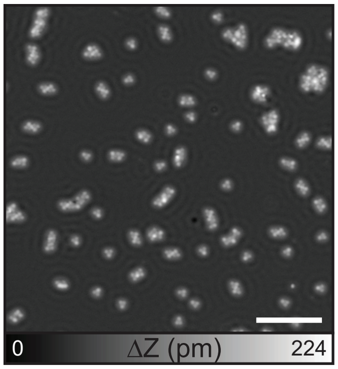

Supplementary Figure 11. | **Hot deposition.** STM topography of the tDBA deposited on the substrate at 200°C. Although the amount of the deposited molecules was the same as that in the RT deposition (at 100°C for 90 sec.), we observed much fewer molecules on Cu(111), indicating that many of the molecules desorbed from the hot substrate. Nevertheless, we found that the ratio of isolated individual molecules increases. In this reaction, the molecule seems to be immediately transformed to the structure shown in Fig. 3c of the main text when they reach to the substrate. Thus, the influence of the Cu adatom, which lowers the reaction temperature and also incorporates into the organometallic bond between the adjacent molecules, is less. Although the product is the same as the one in the reaction by mild annealing, the reaction during hot deposition is presumably caused by the catalysis of the surface Cu atom (not by the Cu adatom), hence avoiding the formation of the organometallic bond. Measurement parameters: $V_{\mathrm{tip}} = -200$ mV and $I = 1$ pA. Scale bar, 10 nm.

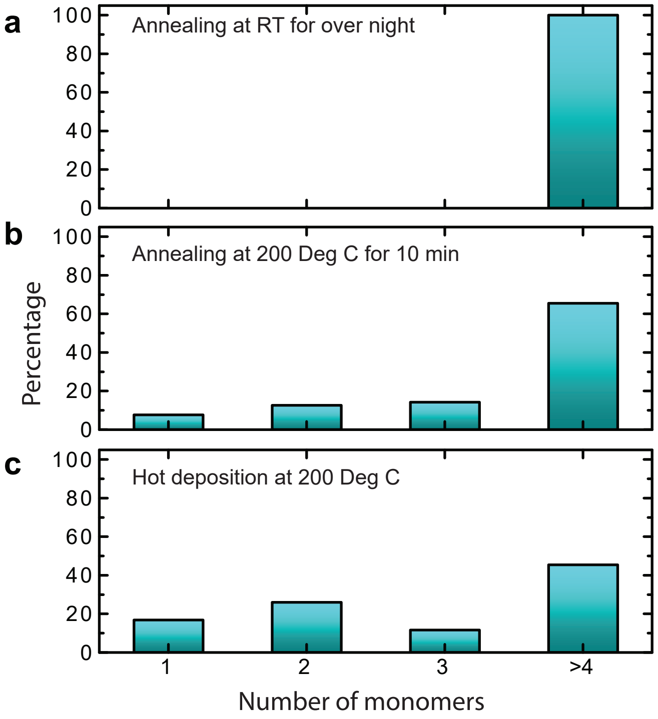

Supplementary Figure 12. | **Histogram of the monomer units in the products.** Histograms of the monomer units in the products for different preparation parameters, showing the influence of the Cu adatom in the on-surface chemical reaction. As shown in Fig. 3 of the main text, Supplementary Fig. 10, and 11, the preparation parameters strongly affect to the product. In order to make a at least a qualitative identification, the products obtained with different parameters were analyzed. The number of the monomer units in the products as a function of preparation were plotted. For the sample annealed at room temperature overnight, no single molecule was observed on Cu(111) and a Cu adatom always plays a role in cleaving the triple bond in the last acetylene. Therefore, all molecules are connected to each other via the organometallic bond. By elevating the temperature, the probability to cleave the triple bond by the catalysis with a surface atom increases. However, since the sample is annealed from low temperature, the formation of the organometallic bond co-exists. In contrast, hot deposition can avoid the formation of the organometallic bond, since the molecules are immediately transformed by the catalysis of the surface atom when they reach to the surface.

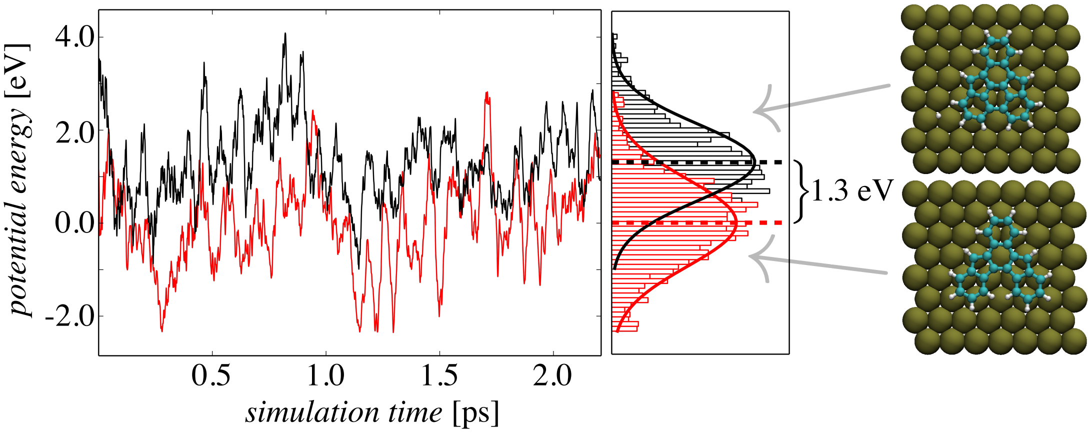

Supplementary Figure 13. | **First-principles molecular dynamics sensitivity testing for the last reaction.** The first one picosecond of the simulation runs was used to thermalise the systems is not considered nor show here. The time-development of the ground-state energies E_o shows that on the average the second-step product is 1.3 eV more favourable than the final product. The E_o of the final structure together with an H_2 molecule is shown in black, the second-step product in red. On the right, the average geometries of the complexes are shown. The averages are taken over the whole trajectories. Note that due to the averaging the H_2 molecule appears as a single H atom.

Supplementary Table

Supplementary Table 1. |**Thermodynamical data for the last reaction.** H_{therm} is enthalpy, S is entropy and G_{therm} is the Gibbs' free energy. All values are calculated at $T = 673.15$ K and $P = 10^{-7}$ Pa. In $G_{\text{therm}}^{\text{scaled}}$ the entropy contribution of the on-surface molecules is scaled by a factor of 0.7 to approximate the constraining effect of the surface (see text). Note: none of the values given here contain ground-state electronic energies.

Molecule	H_{therm} (eV)	S (meV/K)	G_{therm} (eV)	$G_{\text{therm}}^{\text{scaled}}$ (eV)
tDBA 2nd step	10.26	11.849	2.29	4.68
tDBA 3rd step	9.57	11.334	1.94	4.23
H_2	0.49	4.075	-2.26	–

Supplementary Discussion

Estimating the entropic contribution of H and H_2 in the first and last reactions is discussed. In the first and last reaction the number of hydrogens atoms in the molecule changes. Although ground-state electronic energies are in general good qualitative indicators for a physical process (especially at low temperatures), it is the free energy that drives the processes. Here, in the first reaction the change in electronic energy must be large enough to overcome the negative change in entropy caused by capturing two diffusing hydrogen atoms from the surface into the molecular structure. In the last reaction, the entropic contribution to the free energies must overcome the positive change in electronic energy.

We can estimate the entropic contributions (the TS term) using standard statistical physics. In the first reaction we can approximate the on-surface diffusing hydrogens as ideal gas particles; the (transitional) partition function for one such particle is[5]

$$Z_{\text{trans}} = \left(\frac{2\pi M k_B T}{h^2}\right)^{3/2} \frac{k_B T}{P}, \tag{1}$$

which yields for the translational entropy component

$$S_{\text{trans}} = \frac{5}{2}k_B + k_B \ln\left[Z_{\text{trans}}\right]. \tag{2}$$

Here M is the mass of the entity, T the temperature, P the pressure and k_B & h are the Boltzmann & Planck constants, respectively. Using $T = 150$ K and $P = 10^{-7}$ Pa amounts to $TS_{\text{trans}} \approx 0.5$ eV for one hydrogen. For the two hydrogens in the present case this would translate into an entropic penalty of roughly one eV. However, assuming that the on-surface diffusing hydrogens are completely isolated ideal gas particles does arguably overestimate the translational entropy contribution. Recent study investigated the entropies of adsorbed molecules and concluded that the on-surface entropy can be as high as 70% of the gas-phase entropy [6]. Following this, the entropic penalty in the first reaction lowers to about 0.7 eV. The rest of the molecule contributes to the entropy as well – however, the change in entropic contribution of the molecular spine in the reaction is likely to be quite modest. Thus, to a reasonable accuracy the free energy change of the reaction becomes a competition between the change in electronic ground-state energy and the entropic penalty due to the capturing of the additional hydrogens $\Delta G = \Delta H - T\Delta S \approx \Delta E_{\text{o}} - TS_{\text{trans}} \approx -1.4 \text{ eV} + 0.7 \text{ eV} = -0.7$ eV. This analysis indicates that the change in electronic energy is negative enough to overcome the entropic penalty of capturing the two hydrogens, giving a thermodynamic view for the

observed spontaneous reaction.

In the last reaction the DFT calculations reveal that the observed final structure is 1.5 eV higher in ground-state electronic energy than the second-step product (assuming that the two H atoms form H_2 and desorb – the energy difference is 1 eV if the H atoms would adsorb on the surface). However, these are energies at $T = 0$ K but the actual reaction takes place at $T = 673.15$ K. To obtain more insight into the reaction energetics, we run first-principles molecular dynamics simulations for both structures at the reaction temperature. The simulations show that at this temperature a) the two hydrogens indeed form an H_2 molecule which desorbs, b) the final product is still higher in ground-state electronic energy, on the average 1.3 eV higher. This is shown in Supplementary Fig. 13. The simulations also show that the molecular geometries at $T = 673.15$ K do not deform beyond recognition in comparison to the structures obtained at $T = 0$ K (see molecular structures in Supplementary Fig. 13). This suggests that we can use the vacuum geometries of the isolated molecules to estimate the thermal entropic contribution to the free energy change in the last transformation. Again, using statistical physics one can calculate the partition function for polyatomic molecules as [5]

$$Z_{\mathrm{tot}} = \underbrace{e^{-E_{\mathrm{o}}/k_{\mathrm{B}}T}}_{\equiv Z_{\mathrm{elec}}} \underbrace{\left(\frac{2\pi M k_{\mathrm{B}}T}{h^2}\right)^{3/2} \frac{k_{\mathrm{B}}T}{P}}_{\equiv Z_{\mathrm{trans}}} \underbrace{\left(\prod_{i=1}^{N_{\mathrm{vib}}} \frac{e^{-h\nu_i/2k_{\mathrm{B}}T}}{1-e^{-h\nu_i/k_{\mathrm{B}}T}}\right)}_{\equiv Z_{\mathrm{vib}}} \underbrace{\frac{\pi^{1/2}}{\sigma}\left(\frac{8\pi^2 k_{\mathrm{B}}T}{h^2}\right)^{3/2} (I_1 I_2 I_3)^{1/2}}_{\equiv Z_{\mathrm{rot}}} \tag{3}$$

where E_{o} is the ground-state energy, N_{vib} the number of vibrational modes, ν_i the frequency of a vibrational mode i, I_i are the principal moments of inertia for the molecule in question and other symbols are as in the equation (1). Note that for H_2 the rotational partition function simplifies to $Z_{\mathrm{rot}} = 8\pi k_B T I/h^2$. The relevant thermal quantities for the last transition, obtained from Z_{tot} using the standard formula with structures and vibrational frequencies from our DFT calculations, are shown in Supplementary table S1. The transition free energy at $T = 673.15$ K and $P = 10^{-7}$ Pa becomes $\Delta G = \Delta E_{\mathrm{o}}(T = 673.15\ \mathrm{K}) + \Delta G_{\mathrm{therm}} = +1.3\ \mathrm{eV} - 2.6\ \mathrm{eV} = -1.3$ eV. Taking into account the reduction in the entropies of the adsorbed molecules[6] gives for the final free energy change $\Delta G \approx -1.4$ eV. Regardless of the trend in electronic energy, the large enough change in the translational entropy due to the escaping hydrogen molecule renders the reaction favourable in free energy. In the last reaction a new C-C bond is formed (see main

text and e.g. Supplementary Fig. 2). When the system is cooled down from the high reaction temperature to the low measurement temperature of $T = 4.8$ K, the molecule loses its kinetic energy and cannot break the C-C bond and obtain the energetically more favourable geometry.

SUPPLEMENTARY REFERENCES

[1] Boys, S. F., and Bernardi, F. The calculation of small molecular interactions by the differences of separate total energies. Some procedures with reduced errors. *Mol. Phys.*, **1970**, *19*, 553–566.

[2] Hapala, P., Kuchin, G., Wagner, C., Tautz, F.S., Temirov, R. & Jelínek, P. The mechanism of high-resolution STM-AFM imaging with functionalized tips. *Phys. Rev. B*, **90**, 085421 (2014).

[3] Gross, L., Mohn, F., Moll, N., Schuler, B., Criado, A., Guitián, E., Peña, D., Gourdon, A. & Meyer, G. Bond-order discrimination by atomic force microscopy. *Science*, **337**, 1326–1329 (2012).

[4] Moll, N., Schuler, B., Kawai, S., Xu, F., Peng, L., Orita, A., Otera, J., Curioni, A., Neu, M., Repp, J., Meyer, G. & Gross, L. Image distortions of a partially fluorinated hydrocarbon molecule in atomic force microscopy with carbon monoxide terminated tips. *Nano Lett.*, **14**, 6127–6131 (2014).

[5] McQuarrie, D. A. Statistical Thermodynamics. Harper & Row Publishers, Inc., New York, NY, USA (1973).

[6] Campbell, C. T. & Sellers, R. V. The Entropies of Adsorbed Molecules. *J. Am. Chem. Soc.*, **134**, 18109–18115 (2012).
